# Climate change impacts shifting landscape of the dairy industry in Hawai‘i

**DOI:** 10.1093/tas/txac064

**Published:** 2022-05-16

**Authors:** Mandeep Adhikari, Ryan J Longman, Thomas W Giambelluca, C N Lee, Yanghua He

**Affiliations:** Department of Molecular Biosciences and Bioengineering, University of Hawai`i at Mānoa, Honolulu, HI 96822, USA; East West Center, Honolulu, HI 96822, USA; Department of Geography and Environment, University of Hawai`i at Mānoa, Honolulu, HI 96822, USA; Department of Geography and Environment, University of Hawai`i at Mānoa, Honolulu, HI 96822, USA; Water Resource Research Center, University of Hawai`i at Mānoa, Honolulu, HI 96822, USA; Department of Human Nutrition, Food and Animal Sciences, University of Hawai`i at Mānoa, Honolulu, HI 96822, USA; Department of Molecular Biosciences and Bioengineering, University of Hawai`i at Mānoa, Honolulu, HI 96822, USA; Department of Human Nutrition, Food and Animal Sciences, University of Hawai`i at Mānoa, Honolulu, HI 96822, USA

**Keywords:** cattle, climate change, Hawaii, heat stress, rainfall, temperature

## Abstract

Proper knowledge and understanding of climatic variability across different seasons are important in farm management. To learn more about the potential effects of climate change on dairying in Hawaii, we conducted a study on site-specific climate characterization using several variables including rainfall, wind speed (**WS**), solar radiation, and temperature, at two dairy farms located on Hawai`i Island, Hawai`i, in Ookala named “OK DAIRY” and in Upolu Point named “UP DAIRY.” Temperature–humidity index (**THI**) and WS variations in the hottest four months (June to September) were analyzed to determine when critical thresholds that affect animal health are exceeded. Rainfall data were used to estimate the capacity of forage production in 6-mo wet (November to April) and dry (May to October) seasons. Future projections of temperature and rainfall were assessed using mid- and end-century gridded data products for low (RCP 4.5) and high emissions (RCP 8.5) scenarios. Our results showed that the “OK DAIRY” site received higher rainfall than the “UP DAIRY” site, favoring grass growth and forage availability. In addition, the “UP DAIRY” site was more stressful for animals during the summer (THI 69 to 73) than the “OK DAIRY” site (THI 67 to 70) as the THI exceeded the critical threshold of 68, which is conducive for high-lactating cattle. On the “UP DAIRY” site, the THI did not drop below 68 during the summer nights, which created fewer opportunities for cattle to recover from heat stress. Future projections indicated that air temperature would increase 1.3 to 1.8 °C by mid-century and 1.6 to 3.2 °C by the end-century at both farms, and rainfall will increase at the “OK DAIRY” site and decrease at the “UP DAIRY” site by the end-century. The agriculture and livestock industries, particularly the dairy and beef subsectors in Hawai`i, are vulnerable to climate changes as higher temperatures and less rainfall will have adverse effects on cattle. The findings in this study demonstrated how both observed and projected changes in climate support the development of long-term strategies for breeding and holistic livestock management practices to adapt to changing climate conditions.

## INTRODUCTION

Global increase in surface air temperature have been predicted to reach +1.5 °C higher between 2030 and 2052 at the current rate of warming (IPCC, 2018). As a result, the number of extreme heat days (days in a year when the ratio of daily maximum and minimum temperatures exceeds the historical records) was projected to increase in tropical regions (IPCC, 2018). In Hawai‘i, the historical records showed that the mean surface air temperature had increased significantly at +0.052 °C per decade over the past 100 yr (1917 to 2016; McKenzie et al., 2019). The recent four decades (1977 to 2016) have been warmer than other decades in the instrumental records (McKenzie et al., 2019), and warming has primarily attributed to an increase in the minimum temperature (McKenzie et al., 2019). Warming in Hawai‘i is not uniform across the landscape, showing the highest temperature increase at the lowest elevations (McKenzie et al., 2019; Kagawa-Viviani and Giambelluca, 2020). Observed temperature trends at sea level are +0.12 °C per decade (Kagawa-Viviani and Giambelluca, 2020), higher than the global trend of +0.095 to 0.11 °C per decade, as reported by the Inter-governmental Panel on Climate Change (IPCC) in their fifth assessment report (AR5; IPCC, 2018). The temperature in Hawai‘i was projected to have an increasing trend under two emission scenarios (RCP 4.5 and RCP 8.5), specifically, the mid-century would increase ranging from 1.3 to 1.9 °C and the end-century would rise 1.7 to 3.3 °C (Zhang et al., 2016; Elison Timm, 2017).

A previous analysis of rainfall in Hawai‘i showed a 5% to 10% decrease in the wet season (November to April) and a 5% increase in the dry season (May to October) over the past decades (1970 to 2000; Elison Timm and Diaz, 2009). The rainfall was projected to decrease in the future, but the magnitude of the change differs with downscaling products and emission scenarios. It was projected that island-wide rainfall would range from −17% to −19% by the mid-century and from −20% to −28% by the end-century (Elison Timm et al., 2015; Zhang et al., 2016). The largest change in rainfall, −39% decrease, was projected to occur in the dry season at the end-century under the high (RCP8.5) greenhouse gas emission scenario (Elison Timm et al., 2015).

Observed and projected changes to the climate indicate the likelihood of severe impacts on the productivity of crops, livestock, and the global food production system (Hatfield, 2008). According to climate projections, US dairies will experience an annual average temperature increase between 0.8 and 1.3 °C by 2030 (Key et al., 2014). The ambient environment impacts cattle’s performance; therefore, many researchers have defined ambient environment to evaluate production and productivity in changing conditions using one or multiple environmental factors such as temperature–humidity index (**THI**; Thom, 1959; Mader et al., 2010), equivalent temperature index (Baeta et al., 1987), and heat load index (Gaughan et al., 2008). THI is the commonly used indicator to measure the degree of heat stress and combines ambient temperature and relative humidity (NOAA, 1976; Yousef, 1987; Hubbard et al., 1999). Critical ambient temperature for dairy cows is 20 °C, and animals would suffer from heat stress and decline in productivity sharply, if it is above 20 °C (Berman et al., 1985; Johnson, 1987; Igono et al., 1992).

When future warming scenarios are considered, the heat stress on high-yielding dairy cows becomes an increasing concern for the producers (Gauly et al., 2013). Considering the increased milk production of dairy cows and the increased heat loads on the cows, researchers updated the THI limits for the high-lactating cows (Bohmanova et al., 2007; Collier et al., 2011). The threshold THI of 68 for high-lactating cattle and the threshold THI of 72 for low-lactating dairy cattle had been suggested (Collier et al., 2011). In the recently published work, THI values have been redefined as low (<68), moderate (68 to 72), and high (>72) to investigate the impact of heat stress on dairy cattle (M’Hamdi et al., 2021).

Livestock production and its performance is on the left side of the equation: P (Phenotype) = G (Genetics) + E (Environment) + G × E, where environmental factors include feeding, housing, ranch management, and ambient climates. Rainfall and temperature have a significant role in livestock management, particularly in dairy farming, as these factors directly affect feed availability and maintain homeostasis in animals (Rojas-Downing et al., 2017). These changing environmental conditions can increase livestock thermal stress, reduce milk and meat production, and lower animal reproduction rates (Key et al., 2014). The previous data reported that daily milk yield decreases by around 2.2 kg/d when the THI values increase from 65 to 73 for high-lactating dairy cows in the tropics (Collier et al., 2011), and the conception rate of dairy cows get reduced by 4.6% with each unit change in the THI (Hahn, 1995). A sudden increase in air temperature by 1 to 5 °C elevates the risk of cattle mortality with grazing conditions (Howden et al., 2008). Higher temperatures and decreased rainfall reduce the quality and quantity of forage production (Thornton et al., 2009; Polley et al., 2013), ultimately decreasing animal productivity. Higher temperatures and prolonged drought conditions contribute to forage scarcity during dry seasons. The absence of proper adjustment to seasonal variations and strategic adaptation measures to cope with long-term climate change can severely impact small, medium, and even commercial farms.

**Figure 1.**
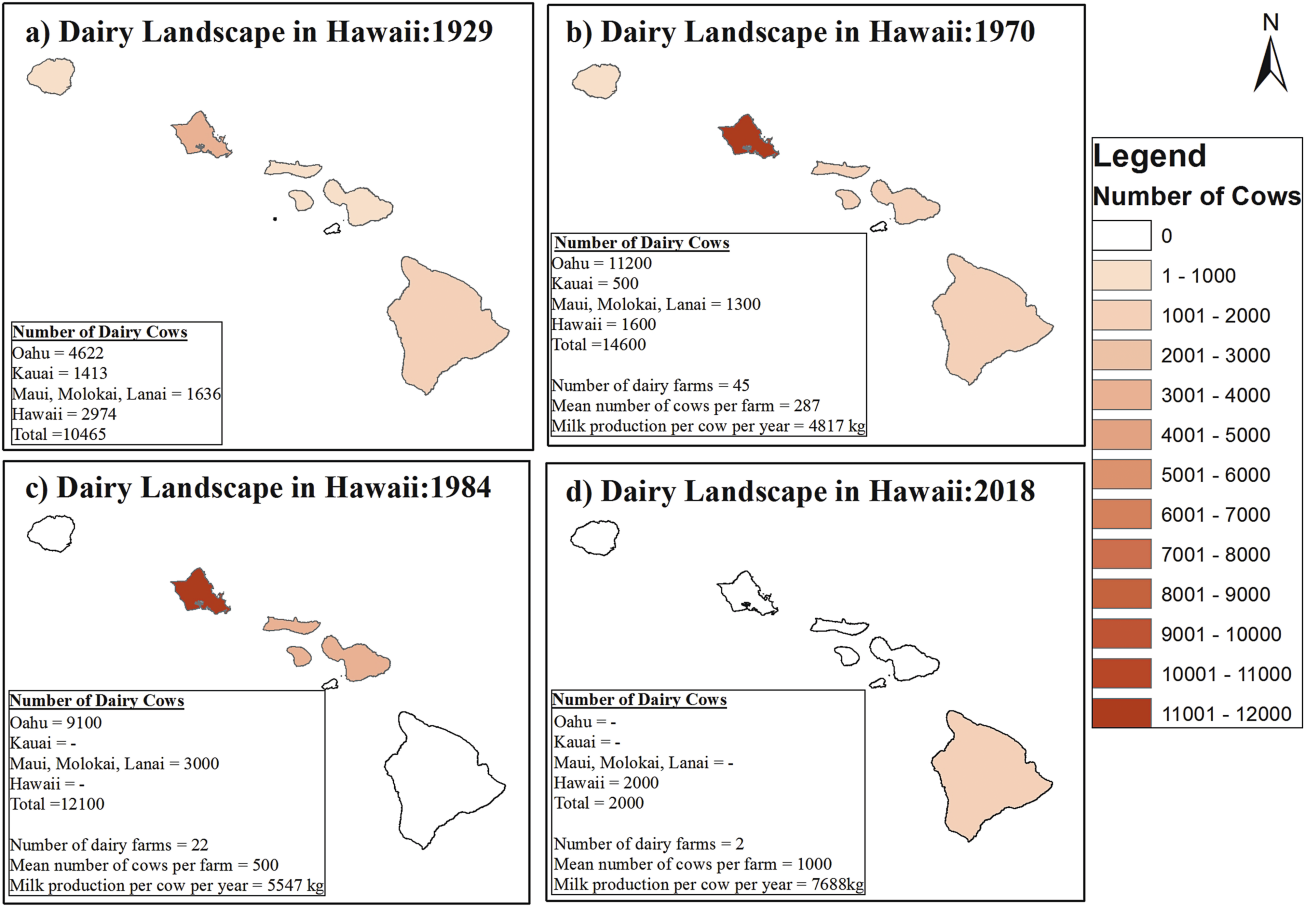
The landscape of dairy farming in Hawai‘i from 1929 to 2018. Landscape of dairy industry in Hawai‘i in (a) 1929; (b) 1970; (c) 1984; (d) 2018.

Hawai`i has a long history of livestock farming. Published articles and reports indicated that dairy farming started in 1793, right after the beef cattle were introduced to the island (Hugh et al., 1986; Lee, 2007). Beef and dairy farming in Hawai`i developed as a single entity during the early phase and continued for several decades until the mid-1800s. Dairy farming was formally separated from the beef industry in 1869, once after operations began at the first commercial dairy farm. By 1919, dairy farms were widespread over all islands in Hawai`i (Hugh et al., 1986). There were approximately 4,622 heads of cattle on Oʻahu and 2,974 on Hawai`i Island by 1929 (Figure 1a). Farming operations and cattle imports continued increasing for the next 5 decades; thus, the total number of cattle heads ranged between 14,600 in the 1970s and 12,100 in 1984 (Figure 1b and c). The island of Oahu led the farming operation and milk production until the 1980s (Figure 1a and c). The pesticide contamination in locally produced milk, widely known as heptachlor incidence, altered dairy farming’s fate in Hawai`i in 1982 (Hugh et al., 1986; Lee, 2007). This incident opened up the market channel for imported milk in Hawai‘i. Consequently, the local products could not compete with the reasonably cheaper milk imported from the mainland of the USA.

The arrival of imported milk in the Islands initiated a sharp decline in cattle operations that continues today. There were 12,000 heads in the 1980s, which dropped down to only 2,000 heads in 2018 (Supplementary Figure S1). Although milk production per cattle has increased from 13 to 17 metric tons during the last three decades, the impact of the 6-fold decrease in cattle number was so strong that a marginal increase in productivity could not compensate for fulfilling the local demand. By 2018, only two dairy farms were functional in Hawai‘i, with 1,400 cattle heads and 600 cattle heads, responsible for supplying 20% of the total demand to the local consumers (Archwamety, 2020). However, the closure of the biggest dairy on the island (located in Ookala, Hawai‘i) in 2019 further increased the dependency of Hawai‘i on imported milk.

The limitation of land and the scarcity of feeds are the hindering forces that oppose the growth/revitalization of the dairy industry in Hawai‘i, which further advances the understanding and application of climates in precision livestock farming. However, future projections of climate change at local farms and how conducive it will be for livestock by the mid- and end-century are yet to be analyzed or discussed. Also, limited research has been conducted on farm-specific microclimatic predictions and their potential implications for developing long-term strategies to cope with the change. Therefore, this research focuses on two farms on the Hawai‘i island to characterize historical climate observations and future climate projections in the contexed dairy farm operations in the State. The overarching objective is to examine the suitability of dairy production in Hawai‘i in the context of future changing climatic conditions for those sites. This article highlights how additional heat stress and forage scarcity due to elevated temperature and reduced rainfall challenge animals’ production and health, forage growth, and ranch management.

## MATERIALS AND METHODS

### Research Sites

**Figure 2.**
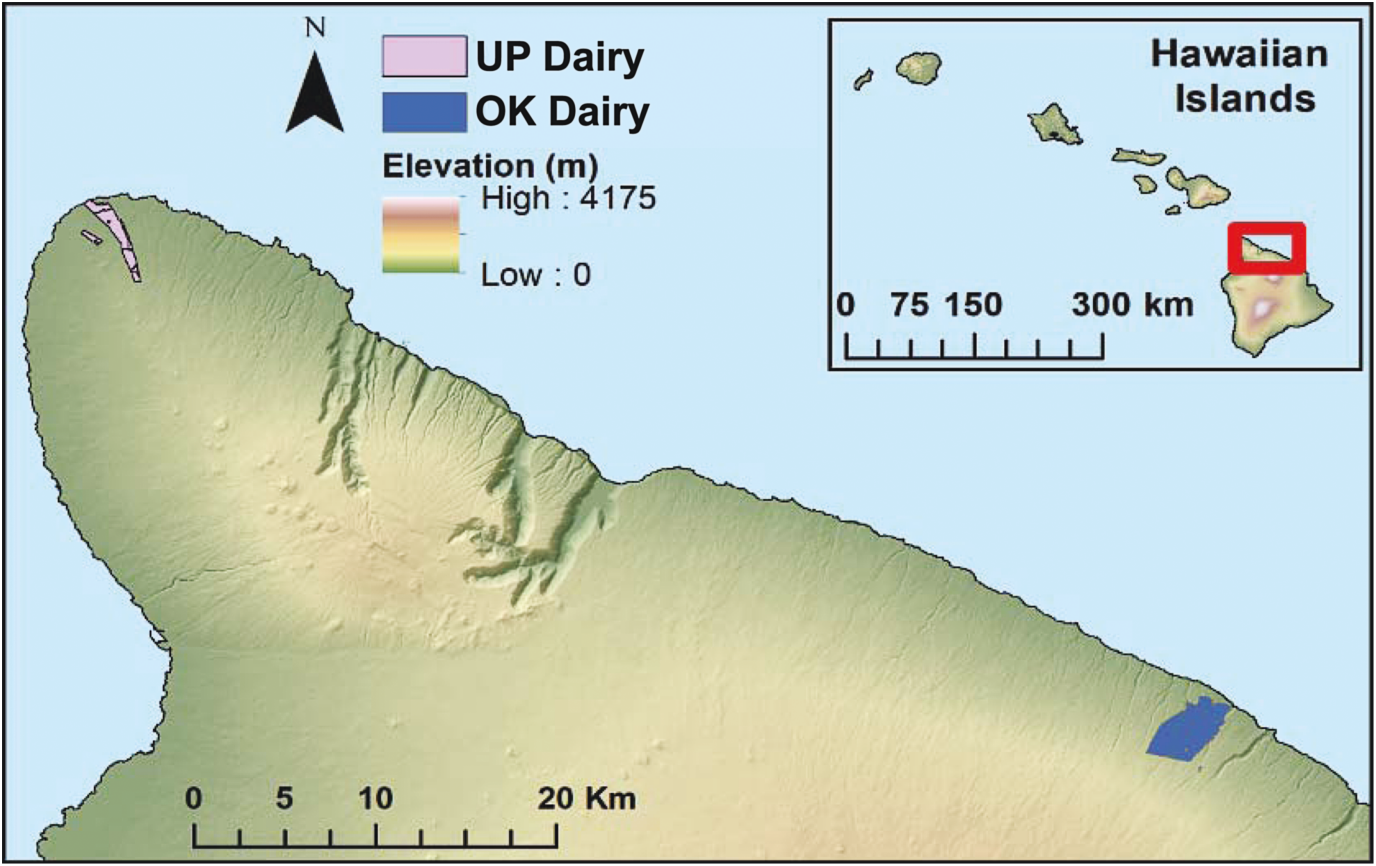
Base map of Hawai‘i showing the location and altitude of the study site.

The dairy farm in Ookala (“OK Dairy” here forward) is located on Hawai‘i island, Hawai‘i, at a mean elevation of 399 m in the range of 47 to 686 m with a total of 2,500 acreages of land. The dairy farm in Upolu Point (“UP Dairy” here forward), also on Hawai‘i island, is located at a mean elevation of 135 m in the range of 0 to 323 m with 880 acreages of land (Figure 2). These two sites Ookala (OK) and Upolu Pont (UP), were potential for a dairy operation, with one functional “UP Dairy” at present, while the “OK Dairy” was closed in 2019 due to environmental concerns (Archwamety, 2020). Despite this closure, the OK site is still considered a suitable site for livestock farming in Hawai‘i. Therefore, it could re-open the dairy operation with appropriate physical adaptation for housing, animal density, and farming practices.

### Climatic Data

A hundred years (1920 to 2019) of monthly rainfall maps (Frazier et al., 2016; Lucas et al. 2022), which were downloaded through the Hawai‘i Climate Data Portal (McLean et al., 2021), were used to characterize rainfall at each site. Monthly rainfall values averaged by 250 m pixels corresponding to each research site and then aggregated to seasonal and annual time steps. Mean-climate data including estimates of rainfall, near-surface air temperature, relative humidity, solar radiations, cloud coverage, soil moistures, evapotranspiration, and soil moisture were obtained from the gridded climate products through the Rainfall Atlas of Hawai‘i (Giambelluca et al., 2013) and the Climate of Hawai‘i (Giambelluca et al., 2014) web portals. A total of 21 years (2000 to 2020) of hourly temperature, relative humidity, and wind speed (**WS**) data were downloaded from the open weather map (https://openweathermap.org/).

### Future Climate Projections

Future rainfall projections were obtained using two available downscaled climate products with two emission scenarios (low emissions RCP 4.5 and high emissions RCP 8.5). Note that downscaling is a method used to relate information obtained from Global Circulation Models to a local scale that takes topography and microclimatic variability into account. In Hawaiʻi, two types of downscaled projections are available: Statistical downscaling (StDs) products (Elison Timm et al., 2015; Elison Timm, 2017) are available for both mid- (2040 to 2070) and end-(2100) century, and dynamical downscaling (DyDs; Zhang et al., 2016) product was applied for the end-century solely. Both StDs and DyDs results have been normalized to a common end-period (2100) and resampled to a consistent spatial resolution (250 m).

### Temperature–Humidity Index

The equation (Mader et al., 2006) to calculate THI is as follows:


$$\begin{array}{l} THI=0.8\times T+RH\times \left(T-14.4\right)+46.4, \end{array}$$

where T is the ambient or dry-bulb temperature in °C, and RH is relative humidity expressed as a proportion (i.e., 75% humidity is expressed as 0.75).

Average temperature and relative humidity data over the past 100 yr (1920 to 2019) were used to calculate monthly THI for the research sites. A THI of 68 is commonly used as a critical threshold to characterize heat stress for high-lactating cattle, while the THI of 72 is used for low-lactating cattle (Collier et al., 2011). The diurnal variation of THI combined with the WS was also analyzed during the four hottest months of the year (June to September).

### Monthly Forage Estimation

Rainfall is positively correlated to forage production (Fay et al., 2008, 2011; Yan et al. 2015). Therefore, the forage production and availability at the two dairy sites were estimated using the Hawai‘i forage production estimator tool (Throne and Hewelett, 2020) based on the monthly rainfall time-series data. The daily forage production quota (**DFPQ**) was used to calculate available forage. The DFPQ is defined as the available dry matter per acre per day per inch of rain. The DFPQ varies from place to place based on soil characteristics, grass type, and climate conditions. Dry matter produced in pasture per month is calculated as follows:


$$\begin{array}{l} Dry\;matter\;production\;per\;month\\ =DFPQ\times monthly\;average\;rainfall\times acres\times 30, \end{array}$$

$$\begin{array}{l} Available\;\;\;drymatter=Dry\;\;\;matter\;\;\;produced/2. \end{array}$$

It is assumed that half of the forage remains in the ground, while the other half is grazed by cattle.

## RESULTS

### Climate Characterization of the Dairy Farms

**Figure 3.**
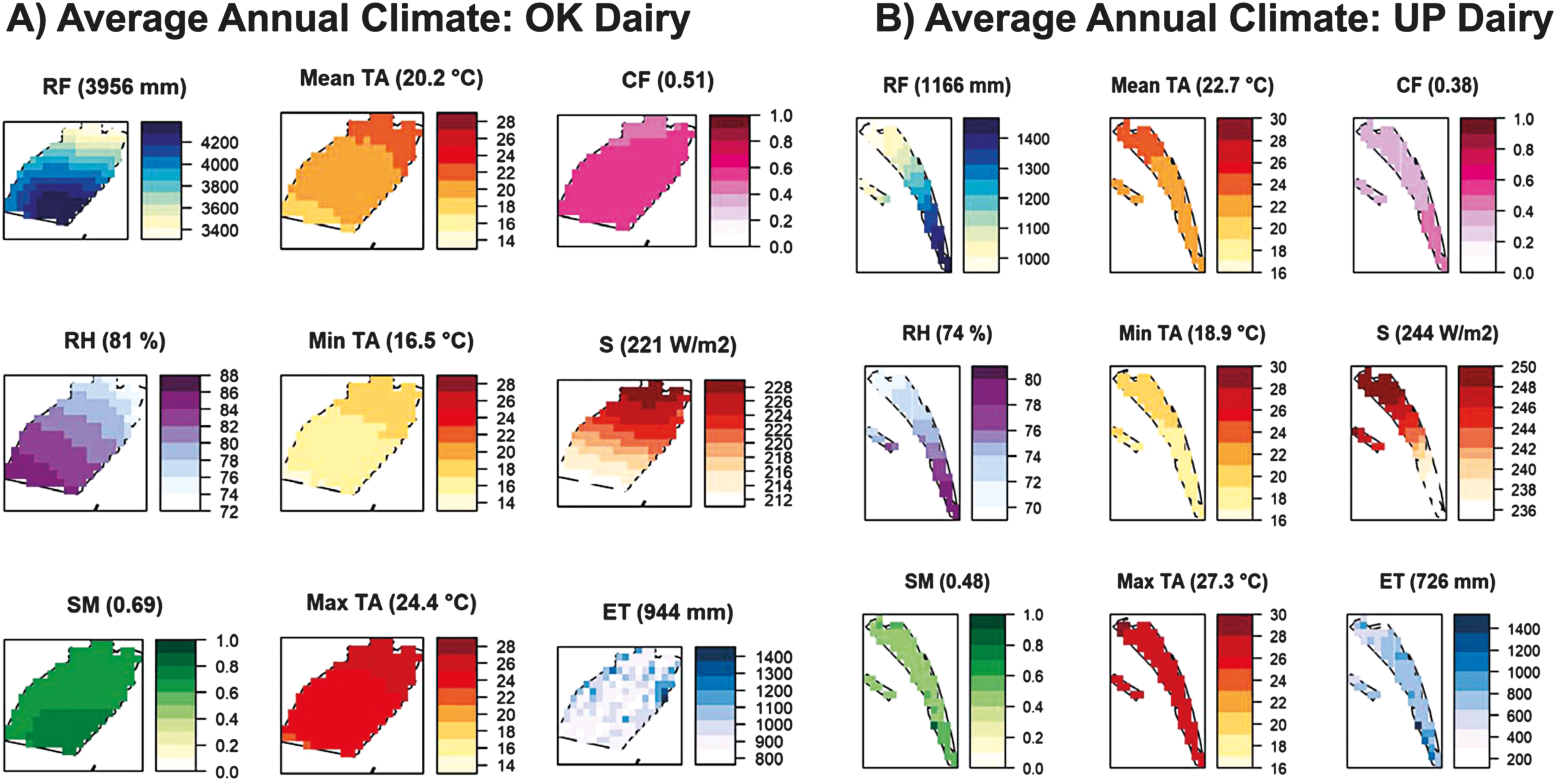
Average annual climatic conditions in two dairy sites. “OK Dairy” site (a), (b) “UP Dairy” site. RF is rainfall (mm), mean TA is average surface air temperature (°C), max TA is average maximum surface air temperature (°C), min TA is average minimum surface air temperature (°C), CF is the cloud fraction, RH is the relative humidity (%), S is the solar radiation (W/m^2^), SM is the soil moisture (ratio), and ET is the evapotranspiration (mm) annual average values shown inside parenthesis.

In Hawai‘i, instep climatic gradients can occur over relatively short distances due to elevation, topography, and orientation to the prevailing winds. Climate characteristics over the past 100 years (1920 to 2019) were analyzed at the “UP Dairy” and “OK Dairy” sites to examine the suitability for dairy cow production now and in the future. The “UP Dairy” and “OK Dairy” sites were shown to have extreme climatic diversity (Figure 3 and Supplementary Table S1), although the distance between them is approximately 65.4 km (Figure 2). The “OK Dairy” was wetter (mean annual RF = 3,956 mm) and more humid (mean RH = 81%) compared with the “UP Dairy” site (mean RF = 1,166 mm; mean RH = 74%). The mean annual rainfall ranged from 3.3 to 4.3 m in the “OK Dairy” site, while the rainfall in the “UP Dairy” site ranged from 1 to 1.5 m. The “UP Dairy” site was relatively warmer where the maximum temperature ranged from 26 to 28 °C compared to the “OK Dairy” site where the maximum temperature ranged from 23 to 26 °C. Since solar radiation and cloud cover directly govern air temperature characteristics, they were also analyzed and showed that the “UP Dairy” site had higher solar radiation (235 to 250 W/m^2^) than the “OK Dairy” site (211 to 229 W/m^2^), while the cloud ratio in “UP Dairy” site was lower (0.42) compared with “OK Dairy” (0.52). Other differences between the two farms included lower soil moisture ratio (0.43 to 0.87) and lower evapotranspiration (112 to 1,553 mm) at the “UP Dairy” site than in the “OK Dairy” site, where the soil moisture ratio ranged from 0.66 to 0.71, and the evapotranspiration ranged from 763 to 1,457 mm. These climatic parameters are directly associated with moisture availability in soil and plants. Although rainfall and temperature are primarily considered in the climatic analysis, the above factors, directly and indirectly, affect the micro-climatic conditions at these locations, thus ultimately influencing livestock and agricultural management practices.

### Rainfall and Temperature Analyses

**Figure 4.**
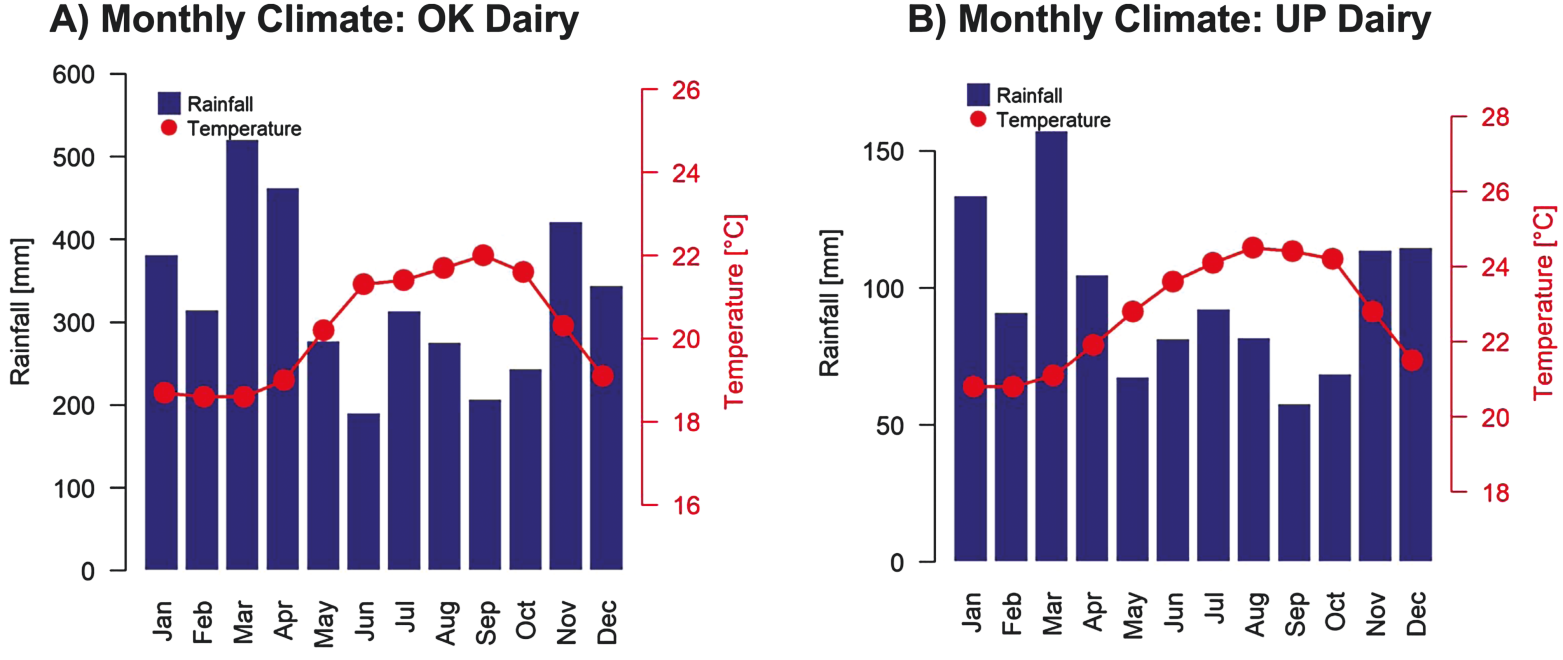
Mean monthly rainfall and temperature (a) “OK Dairy” site, (b) “UP Dairy” site.

A comprehensive analysis of rainfall and temperature was conducted in the two sites with the data from the past 100 yr. Results indicated that average monthly rainfall and temperature patterns varied over the years at the “OK Dairy” site. The highest monthly rainfall at the “OK Dairy” site was received in March (521 mm), while the lowest monthly rainfall was in June (191 mm; Figure 4a, Supplementary Figures S2 and S3). There was a 3.4 °C annual variation in temperature at the “OK Dairy” site with the warmest month in September (22 °C) and the coolest months in February and March (18.6 °C; Supplementary Figure S3). Unlike “OK Dairy,” the “UP Dairy” site had a less pronounced annual rainfall cycle, with the highest monthly rainfall occurring in March (157.3 mm) and the lowest monthly rainfall in September (57.8 mm). Furthermore, the annual temperature for this site fluctuated by 3.7 °C with the warmest month in August (24.5 °C) and the coolest months in January and February (20.8 °C; Figure 4b, Supplementary Figures S2 and S3). In addition, distinct geospatial variabilities of rainfall and temperature throughout the entire year were identified at both dairy sites. The “OK Dairy” site is comparatively larger (2,500 acreages), with most land parcels oriented toward the windward direction, while “UP Dairy” land parcels (880 acreages) have more leeward exposure to the prevailing winds.

### THI and Heat Stress in Cattle

**Figure 5.**
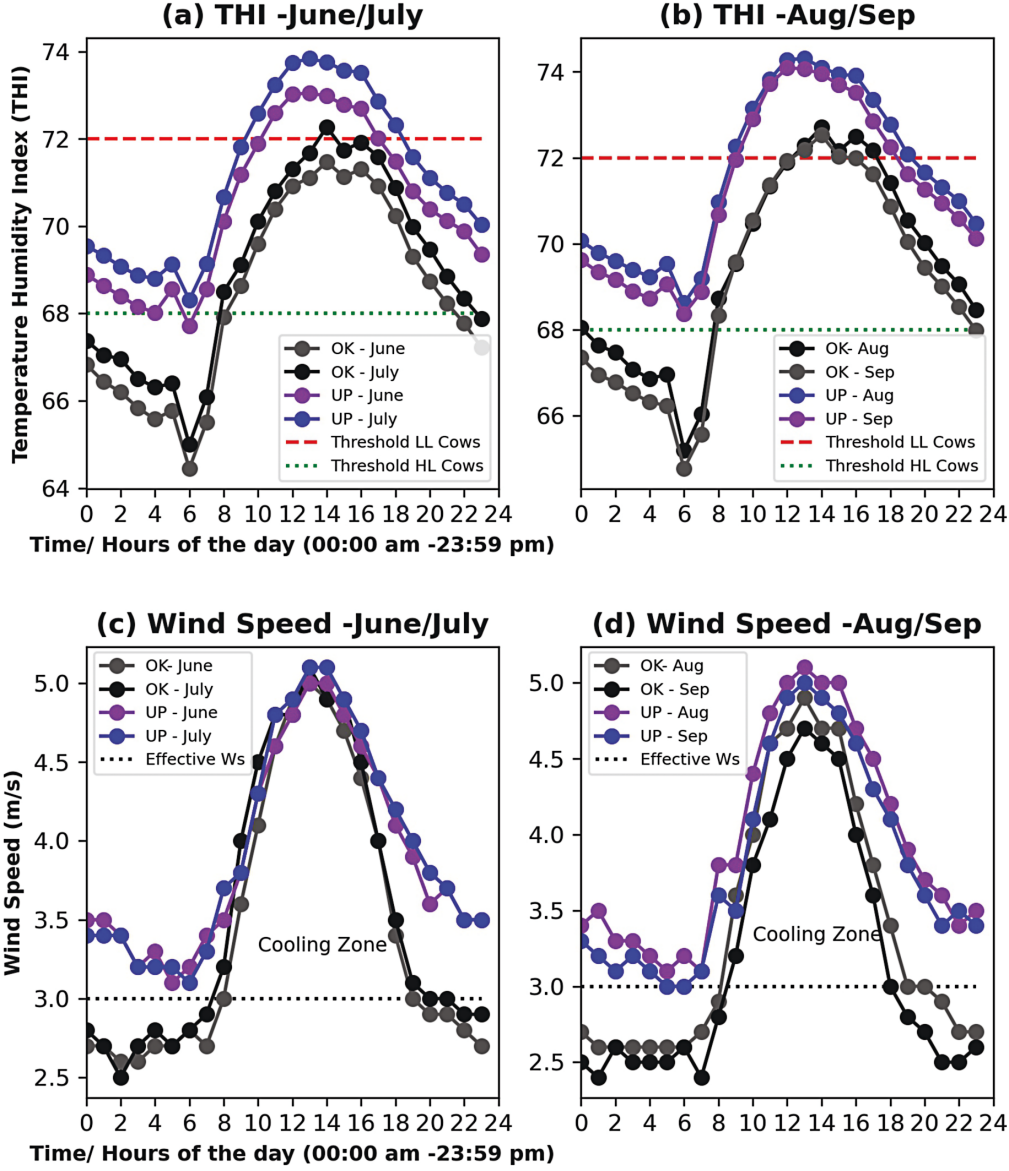
Temperature–humidity index and wind speed across 24 h during the summer season (June to September) using the average data of recent 20 years (2000 to 2020). The dotted horizontal line with the green color above indicates the optimal heat stress threshold for high-lactating dairy cattle. The line with the red color indicates the warning threshold for suffering from heat stress for low-lactating cattle. At the red line, high-lactating cattle suffer even more than low-lactating cattle. The dotted horizontal line with black color indicates the effective wind speed that maintains homeostasis in cattle.

To assess the risk of heat stress on cattle production, monthly THIs were calculated for both locations using the average monthly temperature and humidity data between 1920 and 2019. Results showed that the THI ranged from 64.6 to 70.1 at the “OK Dairy” site, while it ranged from 67.8 to 73.5 at the “UP Dairy” site (Table 1). The four summer months (June to September) at the “OK Dairy” site were not conducive for high-producing dairy cattle (THI > 68). However, the THIs at the “OK Dairy” site never reached 72 (the critical threshold for low-producing cattle) and mostly remained within the range of 67 to 70, indicating favorable conditions for low-producing dairy cattle throughout the year. At the “UP Dairy” site, the THI exceeded 72 throughout the summer and did not drop below the critical threshold of 68 until the middle of the winter (January and February). Therefore, both high- and low-producing animals in the “UP Dairy” site could have experienced more heat stress than those in the “OK Dairy” site. Besides a higher THI, the duration of exposure to high THI reflects the true impact of heat stress on the animals, thus affecting animal health and productivity. Animals have been shown to perform best only if the THI remains below the critical threshold during the relaxation period (Spiers et al., 2001; Lee and Hillman, 2007), and a THI of less than 64 for at least 6 h could reduce the potential harms of heat stress (Igono et al., 1992). Data indicated that the diurnal variation of the THI and WS occurred during the four hottest summer months (June to September), inferring current environmental conditions at both sites (Figure 5). Besides, the THI was the highest from noon to the late afternoon (12:00 p.m. to 04:00 p.m.). The THI began to drop down at night and hit a minimum during the early morning between 4:00 a.m. and 6:00 a.m. The animal’s exposure to stressful heat conditions was calculated for the calendar year, and the results showed that the stressful conditions begin in March and last until December (Supplementary Table S2). The high-producing dairy cows in the “UP Dairy” site were exposed to mild (THI > 68) to moderate (THI > 72) heat stress continuously (14 to 24 h) for several months (April to November). During these periods, THI hardly drops below 68, and therefore the dairy cows in the “UP Dairy” site experience more heat stress in absence of nighttime recovery than in the “OK Dairy” site (Table 1 and Supplementary Table S2).

**Table 1.**
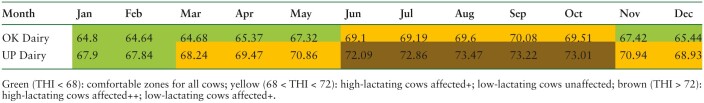
Temperature–humidity index in each month during 1920 to 2019 covered the two dairy farms

It is known that WS is another important factor affecting the animal body’s thermal comfort. Thus, we analyzed how WS varied every hour during the four hottest months (Figure 5c and d) and concluded that the average WS ranged from 2.5 to 5 m/s in the “OK Dairy” site and from 3.2 to 5 m/s in the “UP Dairy” site during these months. WS was lowest during the night ranging between 2.5 and 3 m/s at the “OK Dairy” site and >3 m/s at the “UP Dairy” site. The WS of the “UP Dairy” site during the night was slightly above the threshold cooling level (3 m/s). Therefore, the relatively higher WS at the “UP Dairy” site could potentially dissipate more of the accumulated heat load per stress from the animals than those in the “OK Dairy” site. The WS increased during the day, that is, the WS during the day is greater than WS at night. Also, the WS at the “UP Dairy” site is greater than WS at the “OK Dairy” site, which reflected a positive correlation between the WS and the temperature due to the positive pressure gradient.

### Future Projections of Temperature and Rainfall

The temperature was projected to have an increase of 1.3 to 1.8 °C by the mid-century and an increase of 1.6 to 3.1 °C by the end-century at the “OK Dairy” site (Zhang et al., 2016; Elison Timm, 2017). Similarly, at the “UP Dairy” site, future projections of temperatures implied an increase of 1.3 to 1.8 °C by the mid-century and an increase of 1.6 to 3.1 °C by the end-century (Table 2 and Supplementary Figure S4; Zhang et al., 2016; Elison Timm, 2017). These indicated that both locations would be rapidly warming in the near future. For rainfall projections, both RCP 4.5 and RCP 8.5 were applied. By the mid-century, the mean annual changes of the rainfall would be 3% to 8% at the “OK Dairy” site and −7% to 6% at the “UP Dairy” site, in addition, seasonal changes would be from 3% to 6% during the dry season and from 1.6% to 8% during the wet season at the “OK Dairy” site, while the changes would be −5% during the dry season and −8% to 1.6% during the wet season at the “UP Dairy” site. By the end-century, the rainfall was predicted under the high emission scenario and showed the annual changes of 10% at the “OK Dairy” site and −11% at the “UP Dairy” site. Overall, projections suggested that both hotter and drier conditions are expected for both sites (Table 2).

**Table 2.** Future projections of temperature and rainfall

Location	Changes in the climatic variables	Mid-century change (2040 to 2070)		End-century change (2100)
		Stds (RCP 4.5)	Stds (RCP 8.5)	Stds (RCP 8.5)
OK Dairy	Annual rainfall, %	3	8	10
	Dry season rainfall, %	3	6	19
	Wet season rainfall, %	1.60	8	13
	Annual temperature, °C	1.3	1.8	3.1
UP Dairy	Annual rainfall, %	−7	6	−11
	Dry season rainfall, %	−5.00	−5	−11
	Wet season rainfall, %	−8	1.60	−11
	Annual temperature, °C	1.3	1.8	3.1

StDs (Statistical downscaling) are the climate models for predicting future climates using different representative concentration pathways (RCP). RCP 4.5 considers the low emissions and RCP 8.5 is the high emissions scenario.

### Forage Production and Prediction

Forage production is estimated for both wet and dry seasons based on a total amount of six-month rainfall. Results indicate that the dry season rainfall is 1.5 m, and the wet season rainfall is 2.4 m at the “OK Dairy” site. At the “UP Dairy” site, the dry season average rainfall was 450 mm, and the wet season rainfall was 716 mm (Supplementary Figure S5). As the rain has a direct relationship with vegetation and pasture growth (Owensby et al., 1999; Fay et al., 2011; Yan et al., 2015), therefore, forage production was predicted based on the amount of rainfall received for these two sites (Supplementary Table S3). The results estimated that the monthly forage production per 100 acres of land in the “OK Dairy” site was 69 tons during the wet season and 43 tons during the dry season when other factors remained constant. In contrast, the forage production in the “UP Dairy” site was estimated to be much lower, averaging 20 tons during the wet season and approximately 12 tons during the dry season. Hence, results indicate that the “OK Dairy” site was three times more productive than the “UP Dairy” site in forage production.

**Figure 6.**
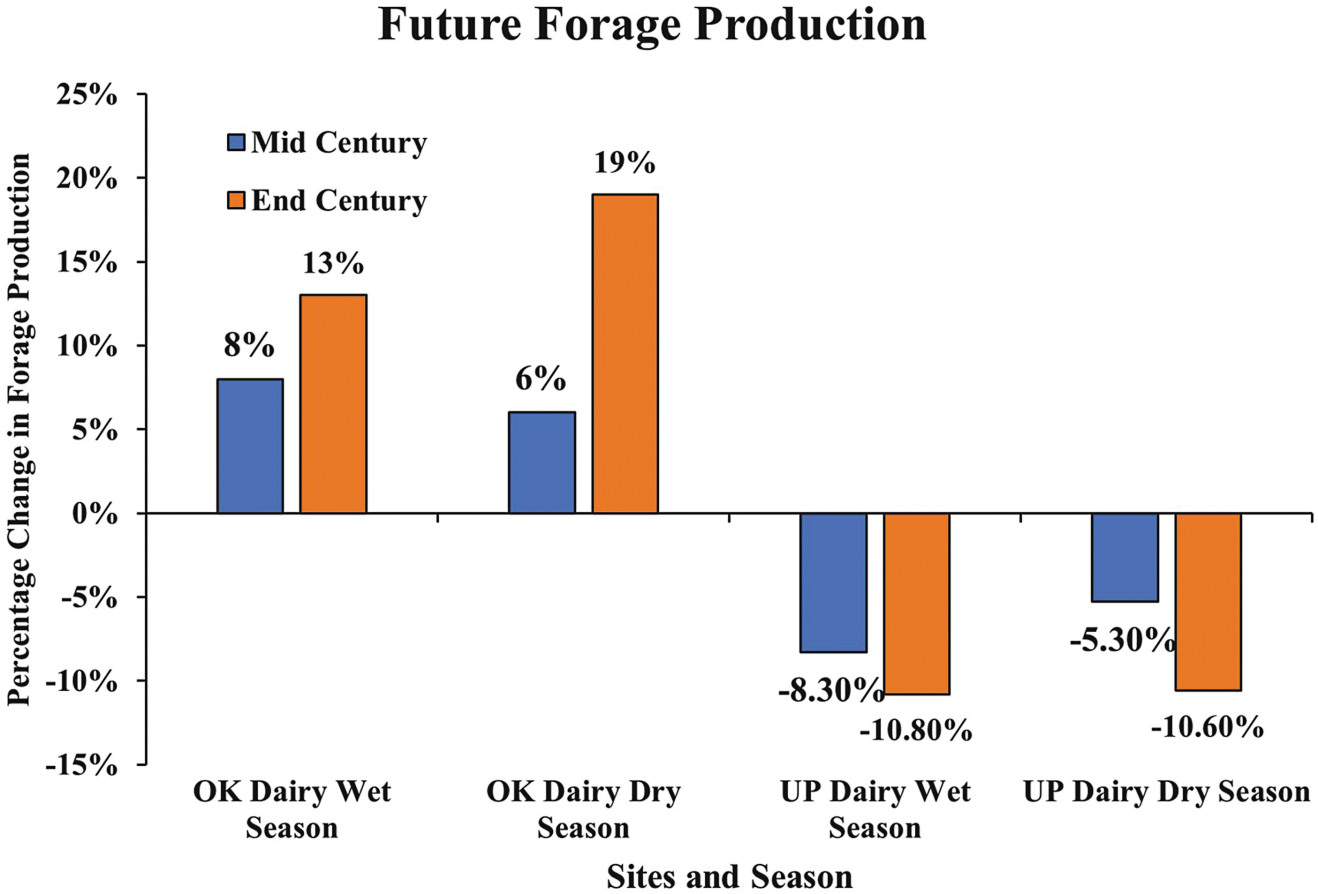
Projected percentage change in forage production at the “OK Dairy” and the “UP Dairy” site by the mid-century and end-century.

Rainfall at the “OK Dairy” site is expected to increase over time, while the “UP Dairy” site can be even dryer by the mid-century and the end-century. Empirical results for future forage production indicated that the monthly forage production in the “OK Dairy” site is projected to increase by 6% to 8% by mid-century and 13% to 19% by the end-century. Whereas, the forage production in the “UP Dairy” site is projected to decrease 5% to 8% by mid-century and 10% to 11% by the end-century (Figure 6, Supplementary Table S3). These projections revealed that the “UP Dairy” site suffers more from forage scarcity, making ranching activities even more difficult in the future unless irrigation is possible. In contrast, “OK Dairy” sites can be even more productive with abundant grass growth in the future.

## DISCUSSION

Livestock farming in Hawai‘i, particularly in lower altitudes, could suffer severe heat stress and forage scarcity due to observed increases in near-surface air-temperature elevated temperatures (McKenzie et al., 2019; Kagawa-Viviani and Giambelluca, 2020) and decreased rainfall (Frazier and Giambelluca, 2017). Understanding the physical mechanisms that govern spatial variation in climates is important in planning the dairy industry, especially in selecting a suitable site conducive to dairy cows. Nighttime warming in the lower elevations (McKenzie et al., 2019; Kagawa-Viviani and Giambelluca, 2020) can make ranch management even more difficult in the future. With the forecast of a 1.3 to 3 °C rise in temperature in Hawai‘i (Zhang et al., 2016; Elison Timm, 2017), the temperature at the “OK Dairy” site is expected to range from 21.5 to 22 °C by the mid-century and from 23.3 to 24 °C by the end-century. At the same time, the expected temperature in the “UP Dairy” site ranges from 24 to 24.5 °C by the mid-century and from 25.8 to 26 °C by the end-century. This gradual and continuous increase in temperature is a severe threat to dairy cows. Genetic merits and desirable adaptation cannot be attained quickly in a few years or decades (Rojas-Downing et al., 2017). The “UP Dairy” site, situated at a lower elevation, is more vulnerable to heat stress than the “OK Dairy” site located at higher elevations. Considering the trend of climate change, perhaps the future distribution of cattle farming can be strategically shifted to more favorable climatic conditions upslope. Therefore, the landscape of dairy farming in Hawai‘i and tropical regions can be biased toward higher elevations in the future. These climatic variations can be appropriately capitalized in dairy farming with smart land management practices and decision-making processes for animal housing, pasture management, herd management, selection of breeds, and altercation in the production and breeding calendar (Rowlinson, 2008; Thornton et al., 2009; Rojas-Downing et al., 2017). For example, relatively dry and cool places are preferred for animal housing to overcome animals’ heat stress. In contrast, hot wet areas are best suited for pastures that favor luxuriant grass growth and quick regeneration. Thus, appropriate land use and management combined with understanding the seasonal variations can reduce the potential operational costs incurred due to environmental stress. Alternatives are the employment of climate modification technologies, for example, fans and misters, alternative energy sources, housing design, housing materials, etc. The cost benefits of such capital improvements must be weighed to ensure the profitability of an operation. In addition, the opportunity to employ biotechnology and the incorporation of genes from animals suitable for the changing climate could be a possibility.

Milk production decreases sharply for every 1 °C increase in air temperature above 21 to 27 °C (Rhoads et al., 2009). Although the number of dairy cows in Hawai‘i is decreasing, the milk production per cow has been increasing during the last 40 yr due to genetic improvement and management practices. These high-producing cows in Hawai‘i are susceptible to heat stress when the THI exceeds critical thresholds. Various literature in the past before the 2000s considered THI of 72 as a critical threshold for all types of cows (Thom, 1959; Mader et al., 2010). However, the heat tolerance in dairy cows and beef cows had notable differences, therefore, modern research includes a different critical threshold for dairy and beef cows. A critical threshold of THI 68 for high-lactating cattle and THI 72 for low-lactating cattle is suggested (Collier et al., 2011). Results showed that the THI in the “UP Dairy” site was higher than in the “OK Dairy” site and only dropped below the animal comfort zone during three mid-winter months (December to March). When the THI exceeded critical thresholds (THI > 68) in the “UP Dairy” site from April to November for 14 to 24 h daily, animals do not get a sufficient (>12 h) nighttime recovery period to counterbalance the additional heat stress of the day. Thus, animals’ health would be severely threatened due to continuous exposure of animals to heat stress for several days. Even mild to moderate heat stress (THI: 68 to 72) can be severely detrimental to animals if THI does not drop below the comfort zone (THI < 68) during the night. Considering the current global trends in warming and future projected increases in temperature in Hawai‘i, critical THI thresholds are projected to be exceeded more frequently throughout the year. In addition, increases in nighttime warming threaten dairy cattle and other domesticated animals like beef cattle, swine, and poultry. Therefore, the selection and breeding of well-adapted animals to the local environment based on their genetic merits can be long-term strategic planning to cope with this inevitable change.

WS plays a vital role in thermal regulation in dairy cows despite the air temperature rising above the critical zone (Akutagawa and Lee, 1992; Spain et al., 1998; Lee and Hillman, 2007). The higher WS is conducive to dissipating animals’ heat stress, and the optimal threshold of WS is 3 m/s (Akutagawa and Lee, 1992), which helps animals regulate thermal homeostasis. As the day progresses, solar radiation intensifies, and grazing cows would experience higher heat stress due to the combined effects of temperature, humidity, and solar radiation from mid-afternoon to sunset (Lee and Hillman, 2007). Cows usually spend about 60% of their time standing and only 14% of their time grazing during the mid-day (Lee and Hillman, 2007). Therefore, animal exposure in pastures during the mid-day in summer is not conducive to their health. Wind plays a vital role in cooling the cows in open fields where active management is challenging to apply. A higher WS of 6 m/s could provide sufficient cooling for mildly heat-stressed lactating cattle (Spain et al., 1998). The wind flow relieves animals from heat stress caused by higher air temperature and relative humidity to a certain degree. Both the “OK Dairy” site and “UP Dairy” site experienced relatively higher wind movement (2.5 to 5 m/s) even during the four extreme months of the year (June to September) during the day, which relieves grazing animals in open pastures or even confined animals in windward flow. However, the WS (~3 m/s) during the night was not convincingly high enough to compensate for the accumulated heat stress of the day for both sites.

The grass production was estimated based on monthly rainfall, and we concluded that the “OK Dairy” site was more productive than the “UP Dairy” site. Complex interactions between the frequency and intensity of rainfall govern the response of grazing land toward rainfall (Fay et al., 2008). Increased temperature with inconsistent rainfall enforces poor quality forage, evidenced by reduced crude protein and total digestible nutrients (Newman et al., 2005; Craine et al., 2010). High temperature accelerates early senescence and desiccation of C3 grasses during the middle and late seasons (Cleland et al., 2006), which causes a scarcity of available forage for livestock which might shift current pasture-based husbandry into grain–grass mixed practice. Increased CO_2_ concentration can positively bolster photosynthetic activity in C3 plants (Parton et al., 2007; Izaurralde et al., 2011) with a commensurate increment in biomass (Owensby et al., 1999). Therefore, future grazing strategies in Hawai‘i and tropical regions might be biased toward selecting and including a higher proportion of C3 species than C4 species for the higher elevation sites where the temperature is relatively cooler. In contrast, the pastures at lower elevations can be biased toward selecting C4 plants more adapted to higher temperatures.

The animals’ pressure on pasture increases with a decrease in rainfall; therefore, the daily dry matter requirement of dairy cows in dry areas can be met by allowing the animals to graze in more acreages of land; however, the land is limited in Hawai‘i. For example, 100 acres of pasture in dry areas can supply forage to fewer cattle than the pastures in wet areas. Ranchers may have to reduce their herds, particularly in the farms that are in dry belts during the dry season, to balance the forage demand and supply. The grass production at the “UP Dairy” site may be severely affected during dry seasons due to a decline in rainfall patterns and elevated temperature by the end-century. In contrast, forage availability in the “OK Dairy” site perhaps is bolstered by increased rainfall. Thus, ranchers in Hawai‘i and tropical regions need to plan early to balance the animal population, cultivate hardy, drought-tolerant grass varieties, and manage groundwater supply to irrigate existing pastures to ensure continuous forage supply. In addition, adaptive response to climate changes involves selecting superior breeds with higher adaptation and implications of breeding strategies to develop genetically resistant breeds against diseases, parasites, and heat stress. Options are already available for dairy cattle as studies had identified the “slick hair gene” in cattle (Olson et al., 2003). Animals with this gene are more heat tolerant (Olson et al., 2003; Davis et al., 2017).

A 3 °C increase in annual temperature in Hawai‘i can significantly increase the THI, resulting in heat stress in dairy cows. Those areas suitable for operating dairy farms at present might not be appropriate after a few decades without proper modifications in housing, management, and other cooling interventions to dissipate additional heat loads. Those dairy farms in the upper critical temperature (20 °C; Johnson, 1987) can turn into heat stress-prone areas by 50 to 70 yr if the current global climate change trend continues. Therefore, site-specific climate studies and interventions on microclimatic modifications, genetic improvement, and holistic adaptation measures become the critical strategy in livestock farming to address the issues and impact of climate changes in Hawaii and tropical regions that are vulnerable to climate change.

## CONCLUSIONS

Understanding the animal and grassland responses to different environmental conditions is essential to successfully implementing strategies to alleviate the adverse effects brought by climate change. Although the “OK Dairy” site is more conducive to dairy cows than the “UP Dairy” site, both locations are vulnerable to the warmer world, where heat stress and periodic forage scarcity are projected to be more common in the future. The temperature rise by the mid- and end-century will likely cause a decline in animal productivity, reproductivity, and increased mortality unless mitigation is made. Above all, results demonstrated that the environment in the “OK Dairy” site is conducive for low-lactating cattle throughout the year, while the potential for animals to experience heat stress at the “UP Dairy” site is much higher. Therefore, to meet the needs of the cattle industry, it is critical to develop strategies in climate change adaptation to address housing, breeding, feeding, and pasture management so that sustainable dairy industries in Hawai‘i can be preserved for future generations. Thus, the findings from this article can be used as a guiding tool to rethink and develop long-term strategies to cope with the inevitable climate change and carry out sustainable farming practices in Hawai‘i and other tropical areas worldwide.

## Supplementary Material

txac064_suppl_Supplementary_Figure_S1

txac064_suppl_Supplementary_Figure_S2

txac064_suppl_Supplementary_Figure_S3

txac064_suppl_Supplementary_Figure_S4

txac064_suppl_Supplementary_Figure_S5

txac064_suppl_Supplementary_Tables

## Data Availability

The data presented here will be retrieved per request.
