# Supplementary figures and images for: Climate change impacts shifting landscape of the dairy industry in Hawai‘i
Source: Transl Anim Sci. 2022 May 16;6(2):txac064. doi: 10.1093/tas/txac064 (PMC9217760; doi:10.1093/tas/txac064)

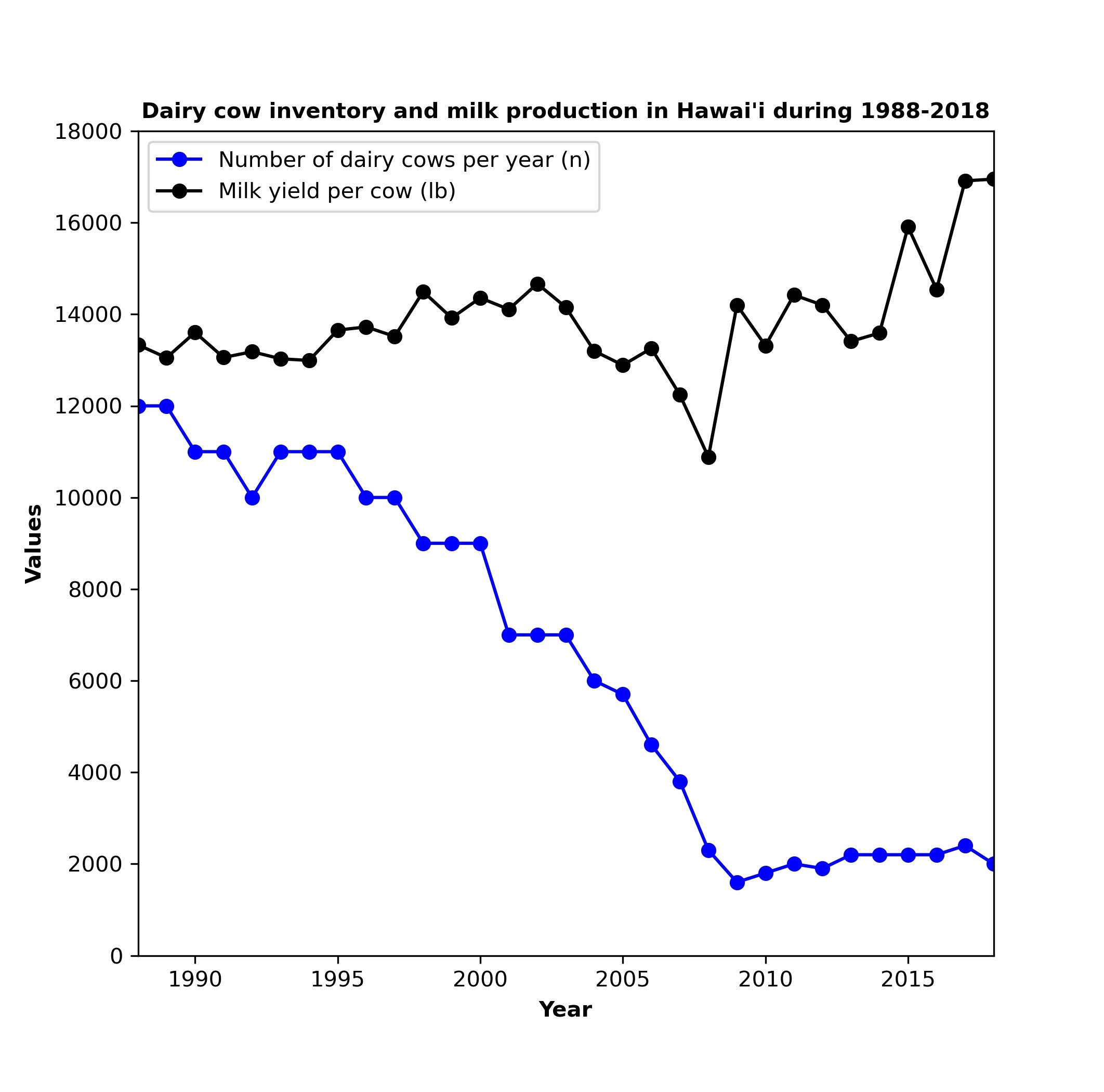

Supplement: txac064_suppl_Supplementary_Figure_S1 [file txac064_suppl_supplementary_figure_s1.jpeg]

## A) Monthly Rainfall: OK Dairy

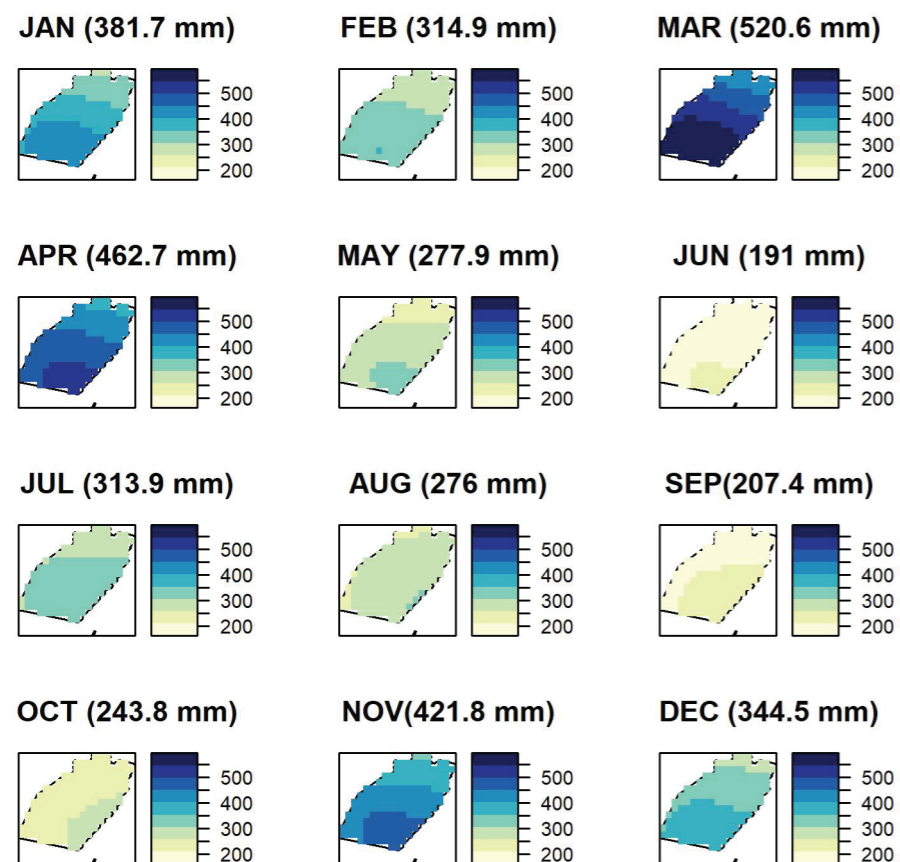

## B) Monthly Rainfall: UP Dairy

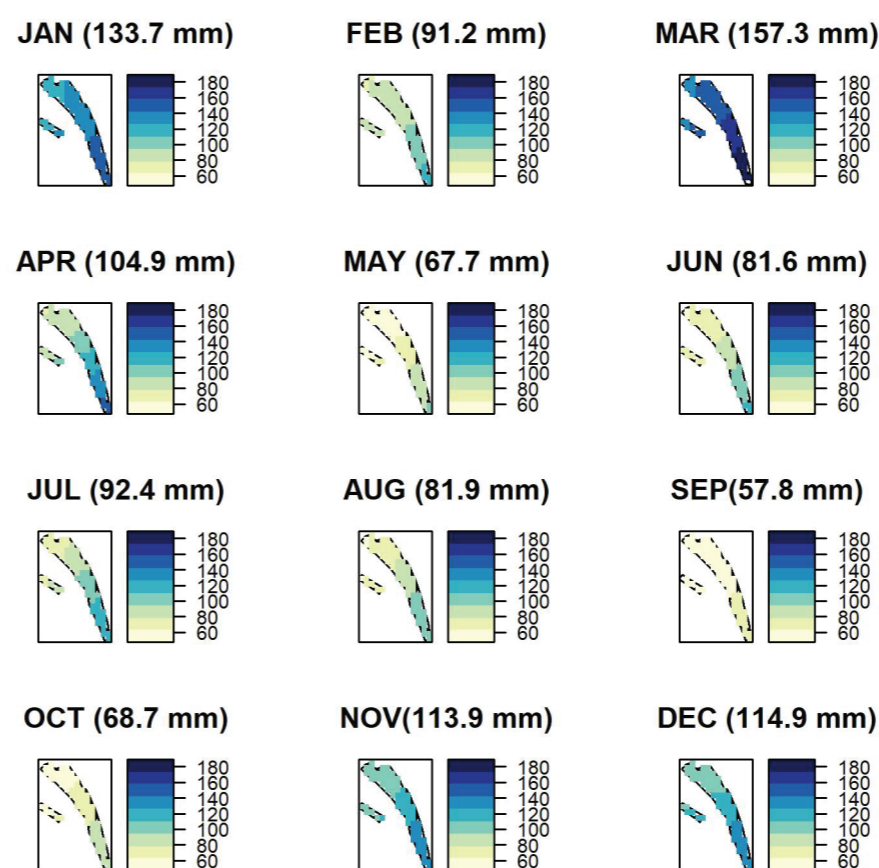

Supplement: txac064_suppl_Supplementary_Figure_S2 [file txac064_suppl_supplementary_figure_s2.pdf]

## A) Monthly Temperature: OK Dairy

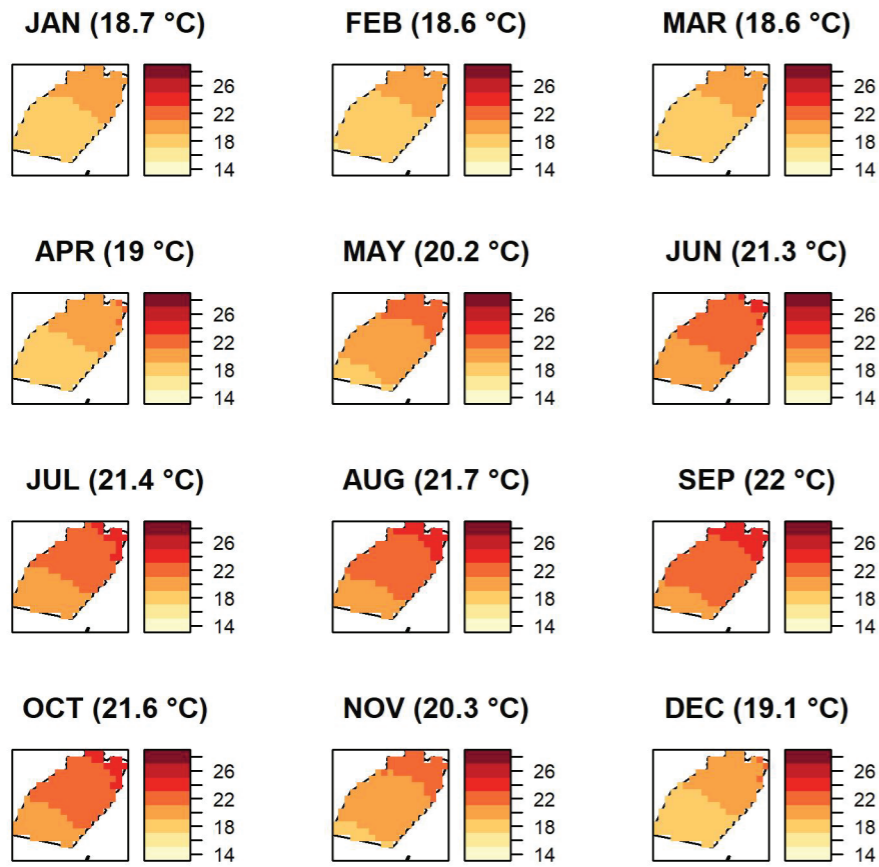

## B) Monthly Temperature: UP Dairy

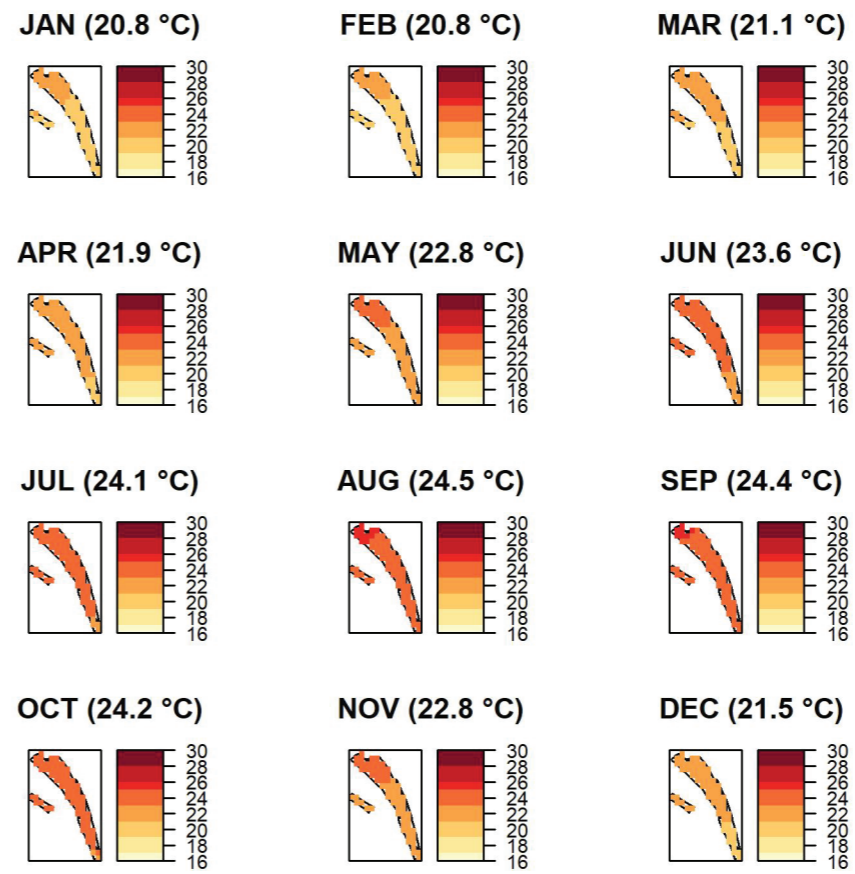

Supplement: txac064_suppl_Supplementary_Figure_S3 [file txac064_suppl_supplementary_figure_s3.pdf]
