# Supplementary material for: Climate change impacts shifting landscape of the dairy industry in Hawai‘i
Source: Transl Anim Sci. 2022 May 16;6(2):txac064. doi: 10.1093/tas/txac064 (PMC9217760; doi:10.1093/tas/txac064)
Supplement: txac064_suppl_Supplementary_Figure_S4 [file txac064_suppl_supplementary_figure_s4.pdf]

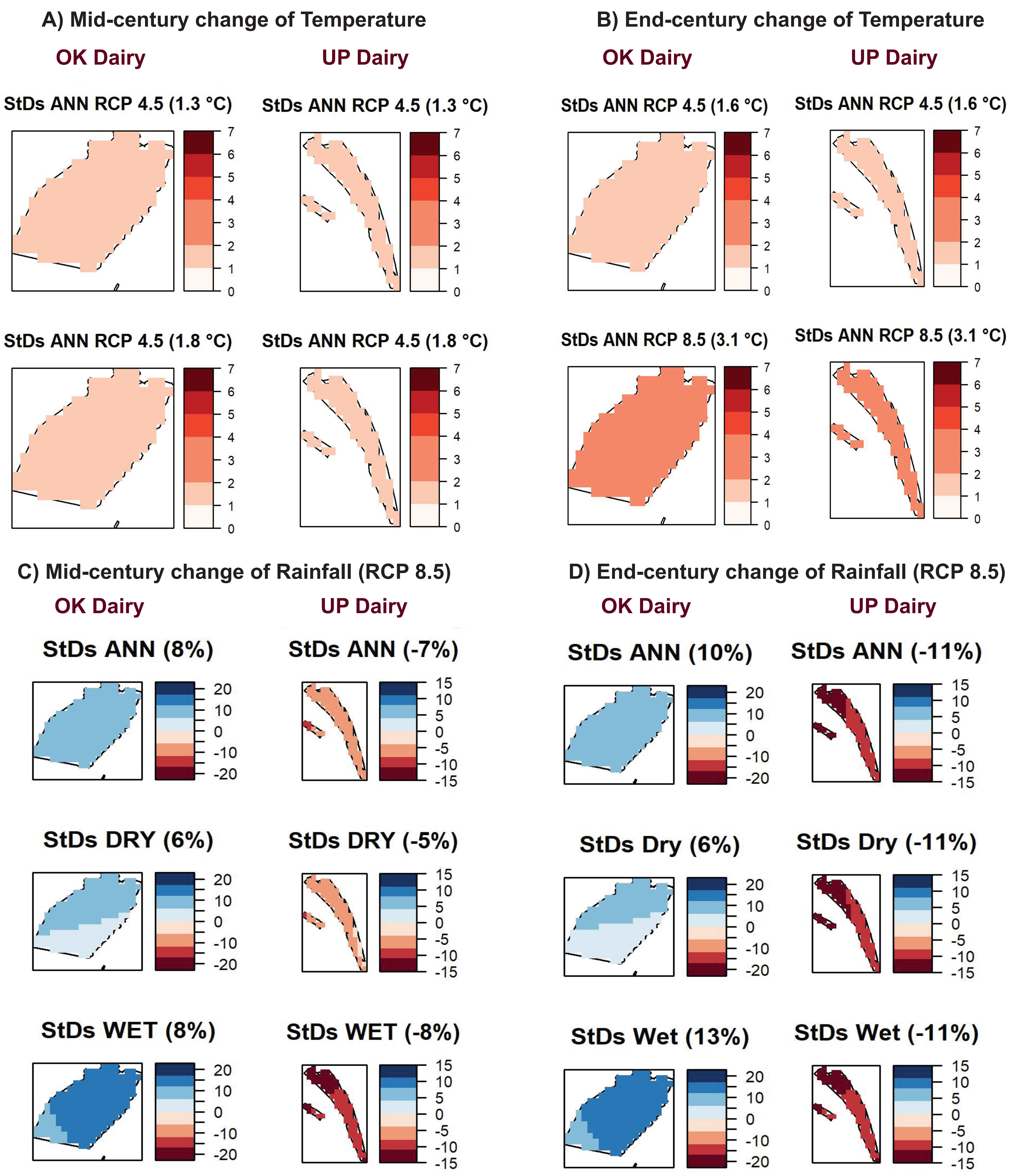

A) Mid-century change of Temperature
OK Dairy
UP Dairy
StDs ANN RCP 4.5 (1.3 °C)
StDs ANN RCP 4.5 (1.3 °C)
StDs ANN RCP 4.5 (1.8 °C)
StDs ANN RCP 4.5 (1.8 °C)
0 1 2 3 4 5 6 7
B) End-century change of Temperature
OK Dairy
UP Dairy
StDs ANN RCP 4.5 (1.6 °C)
StDs ANN RCP 4.5 (1.6 °C)
StDs ANN RCP 8.5 (3.1 °C)
StDs ANN RCP 8.5 (3.1 °C)
C) Mid-century change of Rainfall (RCP 8.5)
OK Dairy
UP Dairy
StDs ANN (8%)
StDs ANN (-7%)
StDs DRY (6%)
StDs DRY (-5%)
StDs WET (8%)
StDs WET (-8%)
20 10 0 -10 -20
15 10 5 0 -5 -10 -15
D) End-century change of Rainfall (RCP 8.5)
OK Dairy
UP Dairy
StDs ANN (10%)
StDs ANN (-11%)
StDs Dry (6%)
StDs Dry (-11%)
StDs Wet (13%)
StDs Wet (-11%)
