# Supplementary material for: Climate change impacts shifting landscape of the dairy industry in Hawai‘i
Source: Transl Anim Sci. 2022 May 16;6(2):txac064. doi: 10.1093/tas/txac064 (PMC9217760; doi:10.1093/tas/txac064)
Supplement: txac064_suppl_Supplementary_Figure_S5 [file txac064_suppl_supplementary_figure_s5.pdf]

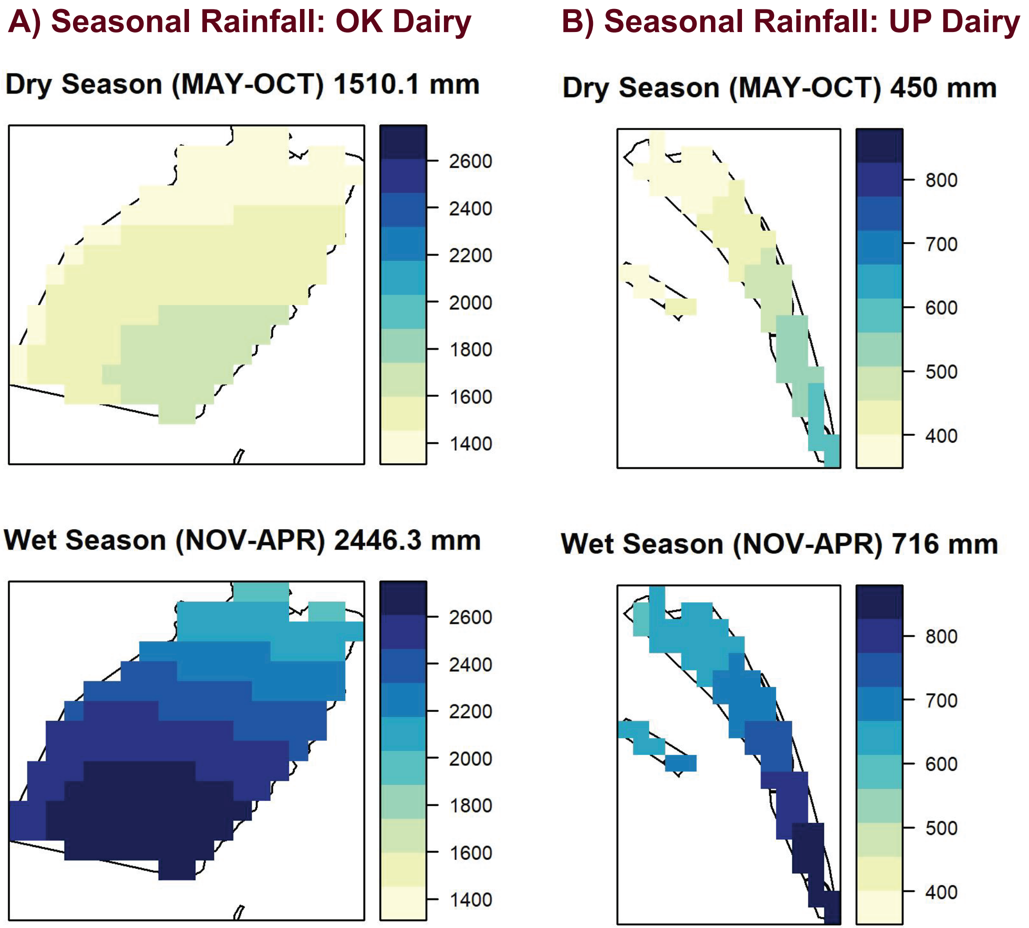
A) Seasonal Rainfall: OK Dairy
Dry Season (MAY-OCT) 1510.1 mm
2600
2400
2200
2000
1800
1600
1400
Wet Season (NOV-APR) 2446.3 mm
2600
2400
2200
2000
1800
1600
1400
B) Seasonal Rainfall: UP Dairy
Dry Season (MAY-OCT) 450 mm
800
700
600
500
400
Wet Season (NOV-APR) 716 mm
800
700
600
500
400
